# LMO2 attenuates tumor growth by targeting the Wnt signaling pathway in breast and colorectal cancer

**DOI:** 10.1038/srep36050

**Published:** 2016-10-25

**Authors:** Ye Liu, Di Huang, Zhaoyang Wang, Chao Wu, Zhao Zhang, Dan Wang, Zongjin Li, Tianhui Zhu, Shuang Yang, Wei Sun

**Affiliations:** 1 Laboratory of Molecular Genetics in School of Medicine, Nankai University, Tianjin, China; 2 Department of Anorectal, Tianjin Union Medical Center, Tianjin, China; 3 Department of Pathology, General Hospital of Tianjin Medical University, Tianjin, China; 4 Laboratory of Stem cells in School of Medicine, Nankai University, Tianjin, China

## Abstract

The proto-oncogene LIM-domain only 2 (*lmo2*) was traditionally considered to be a pivotal transcriptional regulator in hematopoiesis and leukemia. Recently, the cytosolic localization of LMO2 was revealed in multiple epithelial tissues and a variety of solid tumors. However, the function of LMO2 in these epithelia and solid tumors remains largely unclear. The Wnt signaling pathway is a crucial determinant of development, and abnormalities in several key segments of this pathway contribute to oncogenesis. The current study demonstrated that LMO2 participates in the regulation of canonical Wnt signaling in the cytoplasm by binding to Dishevelled-1/2 (DVL-1/2) proteins. These interactions occurred at the PDZ domain of Dishevelled, and LMO2 subsequently attenuated the activation of the key factor β-catenin in the canonical Wnt signaling pathway. Meanwhile, significantly decreased expression of LMO2 was detected in breast and colorectal cancers, and the downregulation of LMO2 in these cells increased cell proliferation and reduced apoptosis. Taken together, the data in this study revealed a novel crosstalk between LMO2 and the Wnt signaling pathway during tumorigenesis and suggested that LMO2 might be a tumor suppressor in certain solid tumors, in contrast to its traditional oncogenic role in the hematopoietic system.

The human LIM-domain only 2 (*lmo2*) gene was first cloned from an acute T lymphocytic leukemia (T-ALL) patient with the (11;14)(p13;q11) translocation^1^, and was revealed to encode a 158-amino acid protein with two-tandem LIM domains as the primary product^2^. Early studies determined that LMO2 is a key regulator of embryonic hematopoiesis and angiogenesis^3^,^4^,^5^ and an oncogene that can trigger T-ALL in both humans and transgenic mouse models^6^,^7^,^8^. In hematopoietic and vascular endothelial cells, LMO2 is located primarily in the cell nucleus and functions as a transcriptional factor but, interestingly, without the ability to bind to DNA directly^9^,^10^. Sporadic recent reports suggested that LMO2 is widely expressed in many other tissues, as well as some solid tumors. Furthermore, LMO2 was located specifically in the cytoplasm in some tissues, particularly in normal and malignant epithelial cells^11^,^12^,^13^. However, the function of LMO2 in these tissues and tumors was rarely reported.

The Wnt signaling pathway plays a pivotal role in regulating embryonic development, including body axis patterning, cell fate specification, and cell migration. Wnt signaling also controls tissue regeneration in adult bone marrow, skin, and intestines^14^. The canonical Wnt pathway is activated by the binding of the ligand Wnt to a Frizzled family receptor, which transmits the signal to Dishevelled proteins (DVLs) inside the cell. This leads to the accumulation and nuclear translocation of β-catenin, where it functions a co-activator of the transcription factor TCF/LEF^15^,^16^. The Wnt signaling pathway is also responsible for tumor development, particularly in cancers of the digestive system, including hepatocellular, pancreatic, gastric and colorectal cancers^17^,^18^,^19^,^20^. Moreover, Wnt signaling also plays pivotal roles in the tumorigenesis of some other tissue-derived cancers, such as breast cancer and melanoma^21^,^22^.

Till now no correlation between LMO2 and Wnt signaling pathway has been reported. Interestingly, our previous investigation on LMO2 interaction partners by pulldown combined with mass spectrum assay revealed a possible protein-protein interaction between LMO2 and Dishevelled-2 proteins, indicating a possible crosslink between LMO2 and the Wnt signaling pathway. Meanwhile, our analysis of the cancer genome atlas (TCGA) online pan-cancer dataset revealed that LMO2 expression was declined in several kinds of solid tumors, particularly in breast and colorectal carcinoma. In this study, we aimed to assess the effects of LMO2 on tumor behavior in breast and colorectal cancers, and investigate the potential role of LMO2 on Wnt signaling pathway in detail. The results revealed that LMO2 could attenuate tumor progression by blocking the Wnt signaling pathway, suggesting a novel tumor suppressor role for LMO2 in contrast to its traditional oncogenic function in the hematopoietic system.

## Results

### LMO2 expression is reduced in various tumors, including breast and colorectal cancer

**Figure 1:**
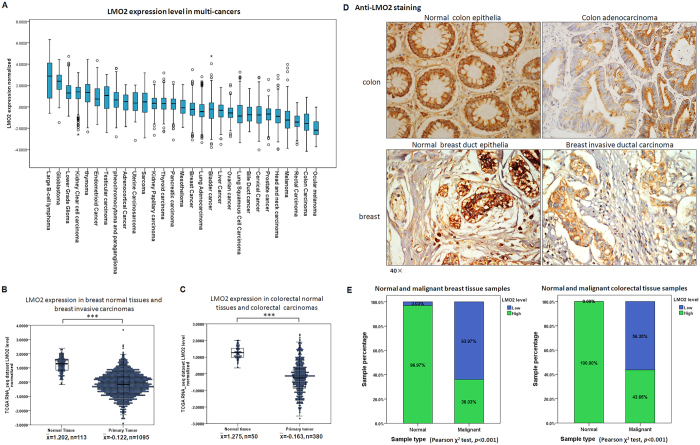
LMO2 expression is reduced in various tumors, including breast and colorectal cancers. (**A**) A box plot showing the medians and ranges of normalized *LMO2* mRNA levels in multiple tumors from the TCGA Pan-cancer dataset. The small circles represent the outliers. (**B**) A two-dimensional scatter plot along with box plot showing the medians and distribution of *LMO2* mRNA levels in normal breast tissue and primary malignant breast tumors in the TCGA breast invasive carcinoma RNA_seq dataset (n = 1208). The means of the relative *LMO2* mRNA level (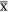) and sample counts are marked in the plot. ***p < 0.001 (Student’s *t*-test). (**C**) A two-dimensional scatter plot and a box plot showing the medians and distribution of *LMO2* mRNA levels in normal colorectal tissues and primary malignant colorectal tumors from the TCGA colorectal carcinoma RNA_seq dataset (n = 430). The mean relative *LMO2* mRNA level (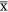) and sample counts are marked in the plot. ***p < 0.001 (Student’s *t*-test). (**D**) Representative images of LMO2 immunohistochemistry from normal breast tissue, primary breast invasive ductal carcinoma, normal colon tissue, and primary colon adenocarcinoma from clinical patient sample sets. Predominantly cytosolic LMO2 staining was observed in most tissues. (**E**) A stacked bar plot showing the distribution of LMO2 expression in normal tissues and primary malignant tumors in 169 breast tissue samples and 140 colorectal tissue samples. The sample count percentage in each group and Pearson χ^2^ test *p*-values are marked in the plots.

To investigate the potential function of LMO2 in tumorigenesis, the Pan-cancer RNA_seq dataset in the TCGA database was analyzed. As shown in Fig. 1A, LMO2 expression varied in a wide range of tumor types; the highest and lowest expression was observed in diffuse large B cell lymphoma (DLBCB) and ocular melanoma, respectively. Notably, significantly reduced LMO2 expression was detected in head and neck, lung, colorectal, breast, prostate, renal, uterine corpus endometrioid, and cervical carcinomas compared with their relevant normal tissues. Conversely, elevated LMO2 expression was observed only in glioblastoma (Table 1). The strongest reduction in LMO2 expression occurred in breast and colorectal cancer, with the strongest statistical significance among the Pan-cancer dataset (Fig. 1B,C, *p* < 0.001).

Next, anti-LMO2 immunohistochemistry was performed in a set of breast tissue samples comprising 33 normal tissues and 136 primary malignant tumors and a set of colorectal tissue samples including 22 normal tissues and 118 primary malignant tumors. The results showed that in general, anti-LMO2 staining was stronger in normal colon epithelia than in colon adenocarcinoma cells, and was stronger in normal breast duct epithelia than in breast invasive ductal carcinoma cells as well (Fig. 1D). Statistical analysis of the staining intensity (Fig. S1A) revealed high expression levels of LMO2 in 97% of breast normal tissues (n = 32) and all colon/rectum normal tissues (n = 22), and this percentage was significantly higher than the corresponding primary malignant tumors (Pearson χ^2^ test, *p* < 0.001, Fig. 1E). These results suggest that LMO2 might function as a tumor suppressor in these solid tumors.

### LMO2 inhibits cell proliferation and increases cisplatin-induced apoptosis in breast and colorectal cancers

**Figure 2:**
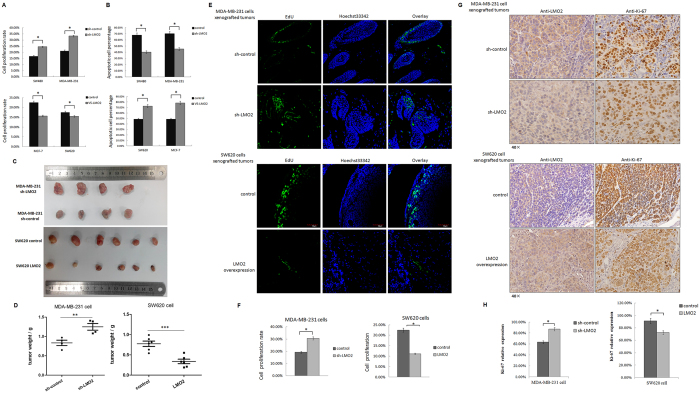
LMO2 inhibits cell proliferation and increases cisplatin-induced apoptosis in breast and colorectal cancers. (**A**) Bar plots indicating the percentage of proliferating cells (EdU-positive/Hoechst 33342-positive cell counts) in the indicated cell lines. The means were calculated using a Cytation^TM^ 3 system from three independent experiments. *p < 0.05 (Student’s *t*-test). (**B**) Bar plots indicating the percentage of apoptotic cells after treatment with 1 μg/ml cisplatin (EdU-positive/Hoechst 33342-positive cell counts) in the indicated cell lines. The mean values were calculated using the Cytation^TM^ 3 system from three independent experiments. *p < 0.05 (Student’s *t*-test). (**C**) Images of xenograft tumors derived from sh-LMO2/sh-control MDA-MB-231 or LMO2 overexpressing/control SW620 cells. (**D**) Scatter plots showing the weight of the xenograft tumors shown in (**C**). **p = 0.0096, ***p = 0.0005 (Student’s *t*-test). (**E**) Representative *in vivo* EdU incorporation assay results from xenograft tumors derived from sh-LMO2/sh-control MDA-MB-231 or LMO2 overexpressing/control SW620 cells. The EdU-positive cells were stained with Fluor-488 and the nuclei were stained with Hoechst 33342. (**F**) Quantification of *in vivo* EdU incorporation assays. The bar plots represent the mean percentage of EdU-positive/Hoechst 33342-positive cell counts of nine independent images of relevant tumors. Images were captured and calculations were made using a Cytation^TM^ 3 system. *p < 0.05 (Student’s *t*-test). (**G**) Images of anti-LMO2 and anti-Ki-67 immunohistochemical staining in consecutive sections of xenograft tumors derived from sh-LMO2/sh-control MDA-MB-231 cells or LMO2 overexpressing/control SW620 cells. (**H**) Bar plots showing the mean percentage of Ki-67-positive cells. The percentages were calculated as the Ki-67-positive/total tumor cell counts from four visual fields for each sample and four or six samples per group. *p < 0.05 (Student’s *t*-test).

LMO2 expression differed among various cancer cell lines. The breast cancer cell line MDA-MB-231 and the colorectal cancer cell line SW480 exhibited high LMO2 expression, whereas MCF-7 breast cancer and SW620 colorectal cancer cells expressed relatively lower levels of LMO2 (Fig. S1B). Then a series of cell lines with overexpression or knock-down of LMO2 were generated (Fig. S2A). Accordingly, knocking down LMO2 (sh-LMO2) in MDA-MB-231 and SW480 cells led to a mean ~15% increased proliferation rate (defined as the EdU-positive cell fraction in the Cell-Light™ EdU DNA cell proliferation assay), whereas the overexpression of LMO2 in MCF-7 and SW620 cells attenuated cell proliferation by 7.5% and 2.4%, respectively (Figs 2A and S2B).

Next, cells were treated with 1 μg/ml cisplatin to induce tumor cell apoptosis. However, sh-LMO2 MDA-MB-231 and sh-LMO2 SW480 cells had a lower proportion of apoptotic cells (~40% vs. 70% in the control cells, as determined by a TUNEL assay). In contrast, the overexpression of LMO2 in MCF-7 and SW620 cells yielded the opposite effects (~75% vs. 50% TUNEL-positive apoptotic cells in LMO2-overexpressing vs. control, respectively; Figs 2B and S2C). These results suggest that the downregulation of LMO2 might accelerate tumorigenesis by promoting cell proliferation and inhibiting apoptosis.

In xenografted severe combined immunodeficiency (SCID) mice, sh-LMO2 MDA-MB-231 cell-derived tumors exhibited faster growth than control tumors, whereas LMO2-overexpressing SW620 cell-derived tumors had a mean smaller volume than control tumors 4 weeks after implantation (Fig. 2C,D). Further, *in vivo* EdU-incorporation assays revealed that more EdU-positive tumor cells were detected in sh-LMO2 MDA-MB-231-derived tumors compared with control cells (30% vs. 19% EdU-positive cells), which is indicative of increased proliferation. In contrast, fewer cancer cells in LMO2-overexpressing SW620 cell-derived tumors were labeled by EdU (12% vs. 22% in control), suggesting a lower proliferating rate (Fig. 2E,F). Similarly, immunohistochemistry staining for Ki-67, which is a nuclear proliferation marker, revealed that sh-LMO2 MDA-MB-231 cell-derived tumors had a higher Ki-67-positive cell percentage compared with control (87% vs. 61%, respectively). In contrast, LMO2-overexpressing SW620 cells yielded the opposite results (74% Ki-67 positive cells vs. 92% in control; Fig. 2G,H).

### LMO2 interacts with Dishevelled-1/2 via their PDZ domains primarily in the cytoplasm

**Figure 3:**
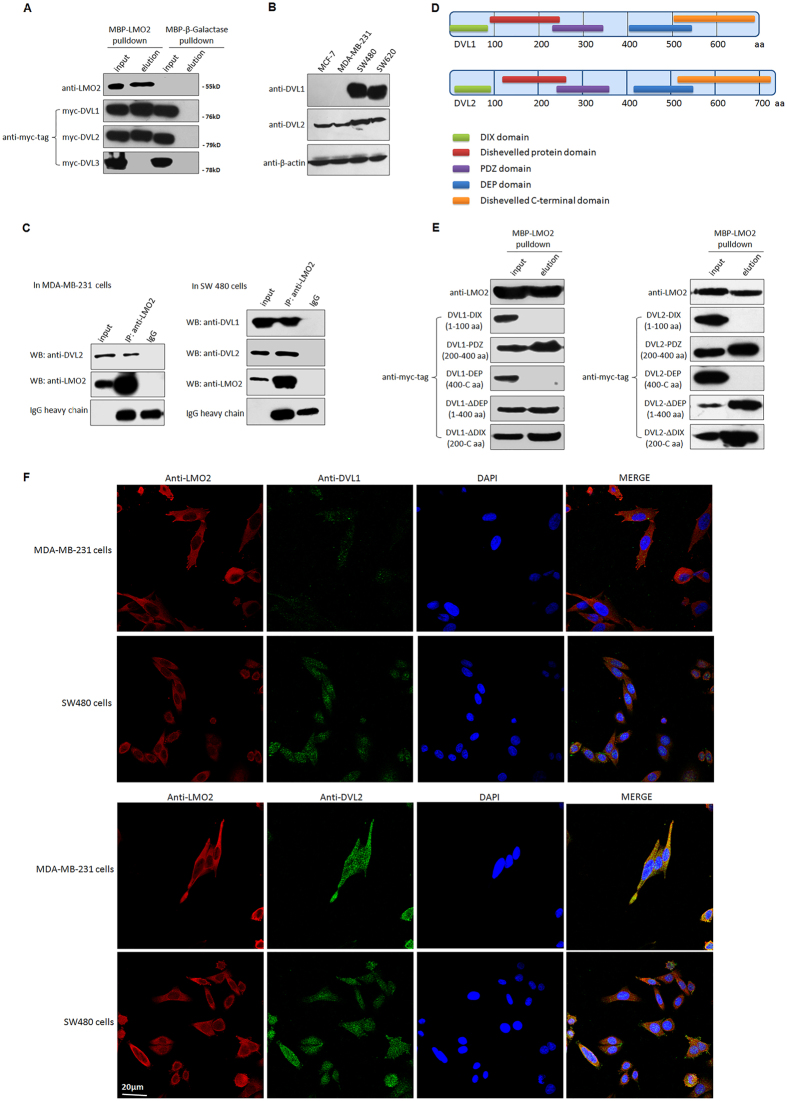
LMO2 interacts with Dishevelled-1 and -2 primarily in the cytoplasm via their PDZ domains. (**A**) MBP-pulldown assay to detect the interaction between LMO2 and DVL-1, -2, and -3. HEK293T cells were used to transiently overexpress Myc-DVL-1, -2, or -3, and cell lysates were incubated with purified recombinant MBP-LMO2 fusion protein or MBP-β-galactase fusion protein (control). Samples were precipitated with amylose resin and immunoblotted with anti-Myc-tag antibodies. A total of 1/20 of the total protein mixture from each sample was used as the input. Anti-LMO2 blots were used to confirm the quality of the experiment. The full-length western blot images are supplied in the Supplementary Info File. (**B**) Western blotting images of DVL-1 and DVL-2 in MCF-7, MDA-MB-231, SW480, and SW620 cell lines; β-actin was used as the loading control. The full-length western blot images are supplied in the Supplementary Info File. (**C**) Co-immunoprecipitation assay to confirm the interaction between endogenous DVL-1 or -2 and LMO2 in MDA-MB-231 or SW480 cells. Cell lysates were immunoprecipitated with anti-LMO2 antibodies and then immunoblotted with anti-DVL1 or -DVL-2 antibodies. One milligram of total protein was used for each experiment, and 1/20 of the total protein from each sample was loaded as the input. The full-length western blot images are supplied in the Supplementary Info File. (**D**) The structure of DVL-1 and -2. The position of the DIX, PDZ, and DEP domains is depicted. (**E**) MBP-pulldown assay to detect the interaction between LMO2 and truncated forms of DVL-1 and -2. The same protocol as described in (**A)** was used. The full-length western blot images are supplied in the Supplementary Info File. (**F**) Representative images of LMO2, DVL-1 and DVL2 immunofluorescence staining in MDA-MB-231 and SW480 cells. The nuclei were stained with DAPI.

Our previous maltose binding protein (MBP)-pulldown and mass spectrum assay suggested a potential interaction between LMO2 and Dishevelled-2 proteins (data not shown). There are three Dishevelled proteins in humans, named DVL-1, -2, and -3^23^. Interactions between the MBP-LMO2 recombinant fusion protein and DVL-1/2, but not MBP-LMO2 and DVL-3, were detected by MBP-pulldown assays (Fig. 3A). Moreover, DVL-1 was expressed at high levels in SW480 and SW620 colorectal cancer cell lines, but only at trace levels in MCF-7 and MDA-MB-231 breast cancer cells. In contrast, DVL-2 was expressed at moderate levels in all these cell lines (Fig. 3B). Subsequent co-immunoprecipitation assays confirmed the binding between endogenous LMO2 and DVL-1/2 in SW480 and MDA-MB-231 cells (Fig. 3C). DVL-1 and DVL-2 share three highly conserved domains: the N-terminal DIX domain, the central PDZ domain, and the C-terminal DEP domain (Fig. 3D)^23^. To further investigate the interaction between LMO2 and DVL-1 and -2, a series of truncated forms of DVL-1 and -2, including the DIX domains (1–100 aa), PDZ domains (200–400 aa), DEP domains (400 C-terminal aa), ΔDEP (1–400 aa), and ΔDIX (200 C-terminal aa), were constructed. MBP-pulldown assays revealed that LMO2 interacted with the truncated forms containing the central PDZ domain (LMO2 bound to PDZ, ΔDEP, and ΔDIX for both DVL-1 and -2), suggesting that the interaction between LMO2 and DVL-1 and -2 was mediated by the PDZ domains (Fig. 3E). Also, anti-LMO2 and anti-DVL1-1/2 immunofluorescence staining in breast and colorectal cancer cells revealed that LMO2 was predominantly located and co-localized with DVL-1/2 in the cytoplasm (Figs 3F and S3A). Co-immunoprecipitation assay in isolated cytosolic and nuclear fraction of MDA-MB-231 and SW480 cells further confirmed that interaction between LMO2 and DVL-1/2 primarily occurred in the cytoplasm, while there was no LMO2 expression in the nuclear fraction in either of the cell lines (Fig. S3B).

### LMO2 blocks the canonical Wnt signaling pathway and reduces the activation of β-catenin in breast and colorectal cancer cells

**Figure 4:**
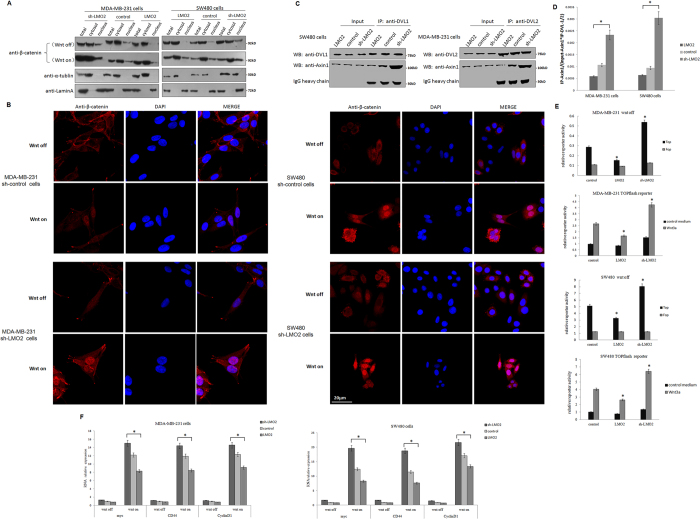
LMO2 blocks the canonical Wnt signaling pathway and reduces the activation of β-catenin in breast and colorectal cancer cells. (**A**) Western blotting for β-catenin in total, cytoplasmic, and nuclear fractions of MDA-MB-231 and SW480 cells under Wnt-off and Wnt-on conditions. α-Tubulin and lamin A were used as the cytoplasmic and nuclear markers, respectively. (**B**) Anti-β-catenin immunofluorescence staining in MDA-MB-231 and SW480 cells under Wnt-off and Wnt-on conditions. β-catenin was stained using a rabbit anti-β-catenin antibody followed by a Fluor-568 secondary fluorescent antibody, and the nuclei were stained with DAPI. (**C**) Co-immunoprecipitation assays to detect the interaction between DVL-1/-2 and Axin1 in LMO2 overexpressing, control, and sh-LMO2 MDA-MB-231/SW480 cells. Cell lysates were immunoprecipitated with anti-DVL-1/2 antibodies and immunoblotted with anti-Axin1 antibodies. One milligram of total protein was used for each IP experiment and 1/20 of the total protein from each sample was used as the input. (**D**) The gray-scale of the immunoblotting bands corresponding to co-immunoprecipitated Axin1 was quantified using ImageJ software. The co-immunoprecipitated Axin1 values were calculated by the formula: IP-Axin1/(input-Axin1*IP-DVL-1/-2), which indicated the amount of Axin1 immunoprecipated by unit amount of DVL-1/-2 in the cell lysate containing unit amount of Axin1 in the corresponding samples. The bars represent the means of 6 independent experiments for each sample, and error bars indicate the standard error. *p < 0.05 (ANOVA followed by Dunnett’s *t*-test vs. control). (**E**) Bar plots showing the TOPflash/FOPflash reporter activity in MDA-MB-231 and SW480 cells under Wnt-off and Wnt-on conditions. The relative luciferase activity was determined after normalization to Renilla. The bars represent the means of three independent experiments, and the error bars represent the standard error. *p < 0.05 (ANOVA followed by Dunnett’s *t-*test vs. control). (**F**) Real-time PCR was used to measure *c-myc, cyclin D1* and *CD44* levels in MDA-MB-231 and SW480 cells under Wnt-off and Wnt-on conditions. The relative expression of each gene was normalized to the housekeeping gene *GAPDH*. The bars represent the means of three independent experiments, and error bars represent the standard error. *p < 0.05 (ANOVA followed by SNK-*q-*test).

Wnt signaling can branch off into different pathways, and each pathway is mediated by a combination of the three Dishevelled protein domains. Activation of the canonical Wnt pathway, which is mediated by the DIX and PDZ domains and causes the accumulation and nuclear translocation of β-catenin, is a major cause of tumorigenesis^23^. Therefore, the influence of LMO2 on β-catenin under basal culture conditions (Wnt-off) or after Wnt3A stimulation (Wnt-on) was investigated. Western blotting and immunofluorescence staining revealed that the knock-down of LMO2 slightly increased the accumulation and nuclear translocation of β-catenin in both breast and colorectal cancer cells in the Wnt-off status. In contrast, the overexpression of LMO2 showed the opposite effects. Moreover, the nuclear translocation of β-catenin increased dramatically after Wnt3A stimulation in these cells (Fig. 4A,B).

The influence of LMO2 on the noncanonical Wnt/planar cell polarity pathway was also examined. Upon Wnt stimulation, Dishevelled proteins use the PDZ and DIX domains to form a complex with Dishevelled-associated activator of morphogenesis 1 (DAAM1) and then activate the small GTPase RhoA, which is a major regulator of cytoskeletal remodeling. However, no prominent change in RhoA activation was detected in several breast and colorectal cancer cell lines under Wnt-on conditions (Fig. S4).

Since the recruitment of Axin1 by the DIX domain of DVLs is the trigger that disassembles the destruction complex and activates β-catenin, the effect of LMO2 on the interaction between DVLs and Axin1 was examined. Co-immunoprecipitation experiments revealed that upon Wnt3A stimulation, knocking down LMO2 significantly increased the amount of Axin1 that co-immunoprecipitated with DVL-1 in SW480 cells, whereas overexpression of LMO2 had the opposite effect. Similarly, increasing amounts of Axin1 were co-immunoprecipitated with DVL-2 in sh-LMO2 MDA-MB-231 cells and less amounts of Axin1 were co-immunoprecipitated with DVL-2 in LMO2-overexpressing MDA-MB-231 cells (Fig. 4C,D). This suggests that interaction between LMO2 and DVL-1/2 at their PDZ domains blocks the recruitment of Axin1 by DVLs and the subsequent release of β-catenin from the destruction complex.

The indirect regulatory effects of LMO2 on the transcription of Wnt signaling downstream targets under Wnt-off and Wnt-on conditions were also detected. In the TOPflash/FOPflash reporter assay, the TOPflsah reporter contained three copies of the wild-type TCF binding site and can respond to Wnt stimulation, whereas the FOPflash reporter contains only the mutant form of the TCF binding site and is used as the negative control. As shown in Fig. 4E, under Wnt-off conditions in both MDA-MB-231 and SW480 cells, sh-LMO2 upregulated TOPflash reporter activity whereas the overexpression of LMO2 exerted the opposite effects. In contrast, there was no significant change in activity at the FOPflash reporter under any conditions tested. This suggests that LMO2 could regulate Wnt signaling activity in cultured cells, even without external Wnt stimulation. After stimulation with Wnt3A (Wnt-on conditions), the TOPflash reporter exhibited significantly increased activity compared with under Wnt-off conditions in both cell lines. Further, consistent with the observations made under Wnt-off conditions, knocking down LMO2 increased the reporter activity whereas overexpressing LMO2 had the opposite effect compared with control in both MDA-MB-231 and SW480 cells. Next, the mRNA expression of several important β-catenin target genes, including *c-myc, cyclin D1*, and *CD44*, was examined by Q-PCR in breast and colorectal cancer cells. As shown in Fig. 4F, there was no significant difference in the transcription of the three genes in sh-LMO2, control, and LMO2-overexpressing MDA-MB-231 and SW480 cells in the Wnt-off position. However, after stimulation with Wnt3A, the transcription of all three genes was upregulated dramatically. Further, sh-LMO2 caused additional upregulation of these target genes, whereas the overexpression of LMO2 reduced gene expression compared with control cells.

### LMO2 and β-catenin expression are negatively correlated in SCID mouse xenograft tumors and clinical patient samples

**Figure 5:**
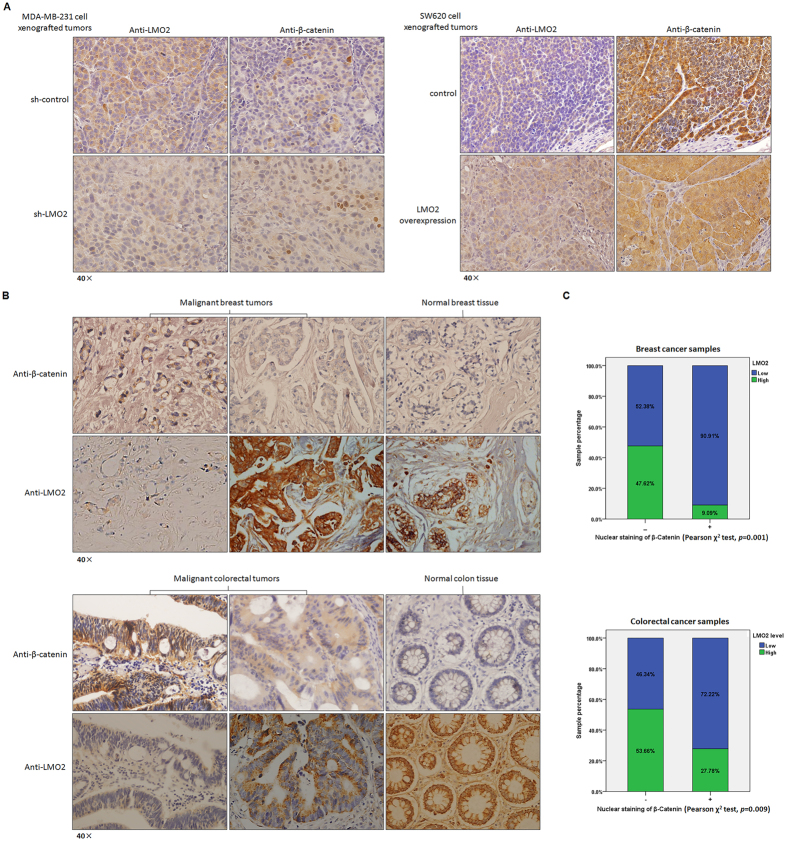
The expression of LMO2 and β-catenin are negatively correlated in SCID mouse xenograft tumors and clinical patient samples. (**A**) Representative immunohistochemical staining of LMO2 and β-catenin in consecutive sections of xenograft tumors derived from sh-LMO2/sh-control MDA-MB-231 cells or LMO2 overexpressing/control SW620 cells. A negative correlation between LMO2 expression and the nuclear localization of β-catenin was revealed. (**B**) Representative immunohistochemical staining of LMO2 and β-catenin in consecutive sections from primary malignant breast/colorectal tumors or normal breast/colon tissues. A negative correlation between LMO2 levels and the nuclear localization of β-catenin was revealed. (**C**) Stacked bar plots showing the distribution of LMO2 expression in nuclear β-catenin-positive or -negative samples among 136 breast tumor and 118 colorectal tumor samples. The sample count percentage in each group and the *p*-values calculated using Pearson’s χ^2^ tests are shown.

Preliminary studies revealed a negative correlation between LMO2 and β-catenin expression in several breast and colorectal cancer cell lines (Fig. S1B). Immunohistochemical staining of consecutive xenograft tumor section derived from sh-LMO2 MDA-MB-231 cells, in which LMO2 expression was lower, revealed obvious β-catenin nuclear staining (which represents the active form of β-catenin). In contrast, β-catenin staining was relatively weak and primarily cytoplasmic in tumors derived from control MDA-MB-231 cells. Similarly, in tumors derived from LMO2-overexpressing SW620 cells higher LMO2 expression, weaker β-catenin staining, and reduced β-catenin nuclear localization were detected compared with control tumors (Fig. 5A).

In clinical sample sets derived from both breast cancer patients and colorectal cancer samples, there was a negative correlation between β-catenin nuclear staining and LMO2 levels (Fig. 5B). In breast cancer samples, 91% of β-catenin nuclear staining-positive samples (21 of 22 samples) revealed low LMO2 expression, whereas only 52% of samples (59 of 114 samples) lacking β-catenin nuclear staining exhibited low LMO2 expression (Pearson χ^2^ test, *p* = 0.001; Fig. 5C). In colorectal cancer, 72% of β-catenin nuclear staining-positive samples (29 of 36 samples) revealed low LMO2 expression, whereas only 46% (38 of 82 samples) of β-catenin nuclear staining-negative samples expressed low levels of LMO2 (Pearson χ^2^ test, *p* = 0.009; Fig. 5C). Also, LMO2 was expressed at consistently high levels whereas low primarily cytoplasmic β-catenin expression was observed in normal breast and colon tissue samples (Fig. 5B). These data further support the hypothesis that high LMO2 levels attenuate the canonical Wnt signaling pathway and tumorigenesis by reducing the activation of β-catenin in breast and colorectal cancer cells.

## Discussion

The Wnt signaling pathway is a pivotal developmental determinant, and abnormalities in several key members of this pathway may contribute to oncogenesis^19^. Notably, mutations in components of the β-catenin degradation complex, such as adenomatous polyposis coli (APC) or β-catenin itself, are common events during tumorigenesis, particularly in colorectal cancer^24^. The current study revealed that LMO2 plays a role in regulating the Wnt signaling pathway by targeting Dishevelled-1 and -2 proteins, which function upstream of the β-catenin degradation complex, and thereby attenuating the activation of β-catenin. In SW480 and SW620 colon cancer cells, despite the presence of APC mutations^25^ and high cellular levels of β-catenin (Fig. S1B), LMO2, which functions in the upstream of the β-catenin degradation complex, downregulated β-catenin activity. Moreover, a previous study revealed that the β-catenin co-transcriptional factor LEF1 directly interacts with LMO2 in the nucleus of DLBCB^26^. However, in the current study, the specific cytosolic localization of LMO2 was observed in both breast and colorectal cancer cells. This is consistent with a previous report^13^ and suggests that LMO2 has a specific cytoplasmic function in these epithelial cells. Accordingly, it could be speculated that the relatively high levels of LMO2 in the cytoplasm may act as a sponge to eliminate inappropriate elevated Wnt activity in normal epithelial cells, whereas this role would be impaired in malignant cells because of the reduced LMO2 levels.

The human *lmo2* gene was classified as an oncogene because of its ability to specifically induce the onset of acute T-lymphocytic leukemia (T-ALL) in hematopoiesis^27^. The oncogenic role of LMO2 was also supported by studies in glioblastoma^28^ and prostate carcinoma^11^. However, in recent years several reports have suggested that LMO2 is a biomarker for good prognosis in DLBCL^29^,^30^,^31^, acute B-lymphocytic leukemia^32^, and pancreatic carcinoma^12^, suggesting that LMO2 could also function as a tumor suppressor. In the current study, analysis of the TCGA Pan-cancer dataset revealed that LMO2 expression was elevated in glioblastoma but reduced in most other cancer types, particularly in epithelial-derived tumors such as breast, colon, rectal, lung, and kidney cancer. These results provide novel evidence that LMO2 might have different roles in epithelia and solid tumors from normal and malignant hematopoietic cells.

The LIM protein superfamily that LMO2 belongs to contains many members with different subcellular localizations and diverse functions; the tandem LIM domains of these proteins mediate a wide variety of protein-protein interactions^33^. Structurally, LMO2 consists of only two tandem LIM domains. This suggests that it might bind to diverse partners from multiple cellular pathways simultaneously; therefore, the overall function of LMO2 in different cell types might depend on its protein interaction profiles and subcellular localization. This might be the molecular basis explaining why LMO2 could function as either an oncogene or a tumor suppressor in different kinds of tumor. Since it functions as a tumor suppressor in epithelial cells, it is possible that the downregulation of LMO2 occurs during the early stages of tumorigenesis in many cancers. One remaining interesting question regarding LMO2 is whether there is a universal mechanism that regulates this process, such as an epigenetic modification. Further investigations into this may help provide novel strategies for cancer therapy by targeting LMO2 and the Wnt signaling pathway in the future.

## Materials and Methods

### Online datasets and statistical analysis

The TCGA Pan-cancer RNA_seq dataset and the relevant clinical information were downloaded from the UCSC Cancer Genomics Browser (https://genome-cancer.ucsc.edu/). The LMO2 expression data from each sample is described in the Supplementary file “Pan_cancer.xls”. Statistical analyses were performed using IBM SPSS Statistics version 20.0 (SPSS Inc., Chicago, IL, USA).

### Clinical samples and immunohistochemistry

All clinical breast and colorectal tissue samples were collected and arrayed by Alenabio Corporation (Xi’an, China) after authorization from the local medical ethics committee. Samples were stained with anti-LMO2, -β-catenin, or -Ki-67 antibodies (1:200 dilution) at 4 °C overnight and then exposed to the appropriate secondary antibodies (1:500 dilution) at room temperature for 1 hour. For LMO2 staining, each sample was scored from 0–5 based on the cytosolic staining intensity; the staining criteria are shown in Fig. S1A. Based on this, samples scoring 0–2 were defined as LMO2 low expression whereas those scoring 3–5 were defined as LMO2 high expression. For β-catenin staining, each sample was grouped based on the nuclear staining signal, which represented activate β-catenin. For Ki-67 staining, the numbers of Ki-67-positive and total cells were counted in each of four visual fields from each xenograft tumor sample under the microscope. The percentage of Ki-67-positive cells was then calculated. All evaluations were performed by two pathologists who were blinded to the experimental groups. Inconsistent results were re-evaluated and confirmed by the third observer.

### Plasmid constructs

Full-length human *Dishevelled-1, -2, -3* and their truncated forms were amplified from peripheral blood cDNA and inserted into pcDNA6B vector with a Myc-tag. The LMO2 coding sequence was inserted into a pMAL vector to express recombinant MBP-LMO2 fusion protein. The lentiviral LMO2 expression vector, LMO2-shRNA, the control lentiviral vector, and the Lenti-Pac™ HIV Expression Packaging Kit were purchased from GeneCopoeia (Rockville, MD, USA). The 293T cell line was used for lentiviral amplification following GeneCopoeia’s instructions.

### Cell culture and transfection

The breast cancer cell lines MDA-MB-231 and MCF-7, the colorectal cancer cell lines SW480 and SW620, and HEK293T cells were obtained from ATCC and cultured in RPMI1640, DMEM, or MEM medium supplied with 10% FBS according to the manufacturer’s instructions (Invitrogen, Austin, TX, USA). HEK293T, MDA-MB-231, or SW480 cells were transfected using Lipofectamine 2000 following the manufacturer’s instructions (Invitrogen). Stable cell strains were selected and maintained by culturing in medium supplied with 2 μg/ml puromycin for 3 days after lentivirus infection. Recombinant Wnt3A proteins were purchased from R&D Systems (Minneapolis, MN, USA) and cells were treated with Wnt3A at a concentration of 200 ng/ml.

### SCID mice xenograft experiments

The experimental protocols were approved by the Animal Care and Use Committee of College of Lifesciences in Nankai University. All experiments were performed in accordance with the relevant guidelines. BALB/c SCID mice were purchased from Charles River Laboratories (Beijing, China) and fed in a specific pathogen-free (SPF) environment. Five-week-old female BALB/c SCID mice were injected with 2 × 10^6^ MDA-MB-231 cells suspended in 50 μl PBS in the right fat pad of the fourth mammary gland. Five-week-old random gender BALB/c SCID mice were injected subcutaneously with 2 × 10^6^ SW620 cells suspended in PBS in a volume of 50 μl in their right groin. Mice were sacrificed 4 weeks after injection and the xenograft tumors were removed, photographed, and fixed in 4% paraformaldehyde for immunohistochemistry staining on 5-μm-thick paraffin sections.

### EdU-labeled cell proliferation and apoptosis assays

Cell-Light™ EdU DNA cell proliferation kit and EdUTP Apollo^®^488 TUNEL cell detection kits were purchased from Guangzhou RiboBio (Guangzhou, China). For *in vitro* assays, 5 × 10^3^ cells were seeded in 96-well plates before assaying. Cell proliferation assays were performed the next day after plating according to the manufacturer’s instruction; cell nuclei were stained with Hoechst 33342. In apoptosis assays, cells were pre-treated with 1 μg/ml cisplatin (Sigma, St. Louis, MO, USA) for 24 h and then analyzed using the kit following the manufacturer’s guidelines. For *in vivo* tumor cell proliferation assays, tumor-bearing mice were intraperitoneally injected with 5 mg/kg EdU reagent for 2 h before dissection. The dissected tumors were analyzed according to the manufacturer’s instructions. The number of Hoechst 33342- and EdU-labeled cells was imaged and counted using a Cytation^TM^ 3 system (BioTek, Winooski, VT, USA). The percentage of proliferating or apoptosis cells was calculated according to the ratio of EdU-positive/Hoechst 33342-positive cell counts.

### Protein extraction, MBP-pulldown, and co-immunoprecipitation assays

Total, cytoplasmic, and nuclear protein extractions were performed using a protein extraction kit (CWBIO, Beijing, China). The protein concentrations were determined using a BCA protein assay kit (Pierce Biotechnology, Rockford, IL, USA). One milligram of total protein from each sample was used for MBP-pulldown or co-immunoprecipitation assays following protocols reported previously^34^. A description of the antibodies used is provided in the Supplementary Information. Immunoblotting bands were subjected to gray-scale quantification using ImageJ software.

### Immunocytofluorescence and confocal microscopy

Immunocytofluorescence was performed in MDA-MB-231 and SW480 cells. A total of 2 × 10^4^ cells were seeded onto cell chamber slides (Corning, Tewksbury, MA, USA) placed in 24-well plates and treated with or without Wnt3A 30 min before assaying. The cells were then fixed with 4% formaldehyde, and stained with anti-DVL-1, anti-DVL-2, anti-β-catenin, or anti-LMO2 antibodies (1:200 dilution) at 4 °C overnight followed by incubation with the appropriate fluorescent secondary antibodies (1:500 dilution) at room temperature for 1 hour. Images were obtained using an FV1000 confocal microscope (Olympus, Center Valley, PA, USA).

### Luciferase reporter assay

The TOPflash/FOPflash reporter system used to measure the response of cells to Wnt stimulation was purchased from Upstate Biotechnology (Waltham, MA, USA). A total of 1 μg TOPflash or FOPflash and Renilla luciferase reporters were co-transfected into cells seeded in 24-well plates at a 5:1 ratio using Lipofectamine2000. Cells were treated with or without Wnt3A for 6 h before assaying, and cells lysed 24 h after transfection. The luciferase activity was then measured using a Dual-Luciferase reporter assay kit (Promega, Madison, WI, USA) and normalized to the Renilla luciferase activity according to the manufacturer’s instructions.

### RNA isolation and real-time PCR

Total RNA was isolated from cells using Trizol reagent (Invitrogen). A total of 1.5 μg total RNA from each sample was used for reverse transcription using M-MLV (Promega). The real-time PCR reagents and primers used to detect *c-myc, cyclin D1*, and *CD44* were purchased from GeneCopoeia (Rockville, MD, USA). Real-time PCR was performed on an ABI PRISM 7000 (Applied Biosystems, Frederick, MD, USA) with the following amplification parameters: 95 °C for 10 min followed by 40 cycles of 95 °C for 10 sec, 60 °C for 20 sec, and 72 °C for 15 sec. Gene expression was normalized to the housekeeping gene *GAPDH*.

## Additional Information

**How to cite this article**: Liu, Y. *et al*. LMO2 attenuates tumor growth by targeting the Wnt signaling pathway in breast and colorectal cancer. *Sci. Rep.* **6**, 36050; doi: 10.1038/srep36050 (2016).

## Supplementary Material

Supplementary Information

Supplementary Information

## Figures and Tables

**Table 1:** LMO2 expression in tumors and their corresponding normal tissues (data from TCGA Pan-cancer dataset).

Tumor type	Mean value of normalized LMO2 expression level (Sample counts)		Statistical significance (*p < 0.05 [Student’s *t*-test])
	Primary tumors	Normal tissues	
Glioblastoma	**2.356 (n = 154)**	**1.201 (n = 5)**	*
Pheochromocytoma and paraganglioma	0.685 (n = 179)	0.569 (n = 3)	
Thymoma	1.233 (n = 119)	0.292 (n = 2)	
Thyroid cancer	0.270 (n = 505)	0.469 (n = 59)	
Sarcoma	0.381 (n = 258)	1.274 (n = 2)	
Head and neck cancer	−0.952 (n = 519)	0.337 (n = 43)	*
Breast cancer	−0.320 (n = 1090)	1.001 (n = 113)	*
Cervical cancer	−0.696 (n = 303)	1.523 (n = 3)	*
Endometrial cancer	0.779 (n = 174)	1.599 (n = 24)	*
Prostate cancer	−0.775 (n = 497)	−0.210 (n = 52)	*
Liver cancer	−0.413 (n = 371)	−0.314 (n = 50)	
Bile duct cancer	−0.743 (n = 36)	−0.411 (n = 9)	
Pancreatic cancer	0.226 (n = 178)	0.826 (n = 4)	
Colon cancer	−1.666 (n = 286)	−0.206 (n = 41)	*
Rectal cancer	−1.508 (n = 94)	−0.110 (n = 9)	*
Lung adenocarcinoma	−0.521 (n = 508)	1.477 (n = 58)	*
Lung squamous cell carcinoma	−0.824 (n = 502)	1.477 (n = 51)	*
Kidney chromophobe	−0.147 (n = 66)	1.681 (n = 25)	*
Kidney clear cell carcinoma	1.328 (n = 529)	1.656 (n = 72)	*
Kidney papillary cell carcinoma	0.187 (n = 290)	1.490 (n = 32)	*
Bladder cancer	−0.336 (n = 407)	0.094 (n = 19)	
Melanoma	−1.470 (n = 103)	0.602 (n = 1)	
