# Supplementary material for: LMO2 attenuates tumor growth by targeting the Wnt signaling pathway in breast and colorectal cancer
Source: Sci Rep. 2016 Oct 25;6:36050. doi: 10.1038/srep36050 (PMC5078767; doi:10.1038/srep36050)

LMO2 attenuates tumor growth by targeting the Wnt signaling pathway in breast and colorectal cancer

Ye Liu[1], Di Huang[1], Zhaoyang Wang[1], Chao Wu[1], Zhao Zhang[2], Dan Wang[3], Zongjin Li[4], Tianhui Zhu[1], Shuang Yang[1], Wei Sun[1]*

[1] Laboratory of Molecular Genetics in School of Medicine, Nankai University, Tianjin, China

[2] Department of Anorectal, Tianjin Union Medical Center, Tianjin, China

[3] Department of Pathology, General Hospital of Tianjin Medical University, Tianjin, China

[4] Laboratory of Stem cells in School of Medicine, Nankai University, Tianjin, China

*Corresponding author: Dr. Wei Sun at Laboratory of Molecular Genetics, School of Medicine, Nankai University, Tianjin, P.R. China.

Tel/Fax: 86-22-23504478.

Email address: sunweibio@nankai.edu.cn.

Supplementary Information

1. Supplementary methods

1.1. RhoA activity detection assay

Assays were performed according to the instructions provided with the RhoA Activation Assay kit (Abcam, Cambridge, UK). Cells were cultured to approximately 8–~90% confluence and stimulated with 200 ng/ml Wnt3A for 15 min before harvesting. Whole cell lysates were collected, aliquoted into tubes containing 1 mg of total cellular protein and adjusted to a volume of 1 ml with 1 × assay buffer. Then, 1 μl anti-active RhoA antibodies and 20 μl protein A/G agarose beads were added to each tube for the pulldown assays. After pulldown, western blotting was performed with anti-RhoA antibodies.

2. Primer information

**Primer names**	**Sequences 5´–3´**	**Restriction site**
LMO2-forward	AATGCGGGTGAAAGACAAAG	–
LMO2-reverse	CCCCAAAGTGCCTAAGAGTG	–
GAPDH-forward	TGAAGGTCGGTGTGAACGGAT	–
GAPDH-reverse	CATGTAGGCCATGA GGTCCACCAC	–
Dvl1 F1	GTCGAATTCGGATGGCGGAGACCAAAATC	*Eco*RI
Dvl1 F2	GTCGAATTCGGGACCTGCCCCCACCCCTT	*Eco*RI
Dvl1 F3	GTCGAATTCGGAGCCGGCTGAGCAGCTCC	*Eco*RI
Dvl1 R1	GTCGGTACCTCACATGATGTCCACAAAGAA	*Kpn*I
Dvl1 R2	GTCGGTACCTCAGCCAGGCACTGAGCTGGT	KpnI

Dvl2 F1	GTCGAATTCGGATGGCGGGTAGCAGCACTG	*Eco*RI
Dvl2 F2	GTCGAATTCGGCCTCCAGTCCATGAGCCTC	*Eco*RI
Dvl2 F3	GTCGAATTCGGTCATCCTTCAGCAGCGTCA	*Eco*RI
Dvl2 R1	GTCGGTACCCTACATAACATCCACAAAGAACTCG	*Kpn*I
Dvl2 R2	GTCGGTACCCTAGCTCATGGAGGAGGAACCTG	*Kpn*I

Wnt signaling pathway downstream genes (purchased from GeneCopoeia)

Genes	product number
CD44	HQP022972
Cyclin D1	HQP016204
c-myc	HQP011597

3. Antibody information

Product name	Catalog number	Company	Application[1]
Anti-rabbit IgG	7074	CST	WB
Anti-mouse IgG	7076	CST	WB
Anti-α-Tubulin	ab108629	Abcam	WB
Anti-Lamin A antibody	ab26300	Abcam	WB
Anti-β-actin Antibody	21338	SAB	WB
Myc-Tag（9B11）Mouse mAb	2276	CST	WB
Anti-Disheveled 2 antibody	ab22616	Abcam	WB
Anti-Disheveled 2 antibody	3216	CST	IP, IF

Anti-Disheveled/Dvl1 antibody	ab170694	Abcam	WB, IP, IF
Anti-β-Catenin antibody	ab32572	Abcam	WB
Anti-β-Catenin antibody	8480	CST	IF, IHC
Anti-Axin1 antibody	2087	CST	IP
Anti-Ki-67 antibody	ZM-0166	ZSGB-BIO	IHC
Anti-LMO2 antibody[1A9-3B11]	ab81988	Abcam	WB, IF, IHC
Anti-LMO2 antibody-ChIP Grade	ab72841	Abcam	IP
Alexa fluor 546 donkey anti-rabbit IgG	A10040	Invitrogen	IF
Alexa fluor 568 donkey anti-mouse IgG	A10037	Invitrogen	IF
Alexa fluor 488 goat anti-rabbit IgG	A11008	Invitrogen	IF
RhoA Activation Assay kit	Ab173237	Abcam	RhoA activation assays

1. abbreviations: WB, Western blot; IP, Immunoprecipitation; IF, Immunofluorescence; IHC, Immunohistochemistry

Supplementary Figures and Figure legends

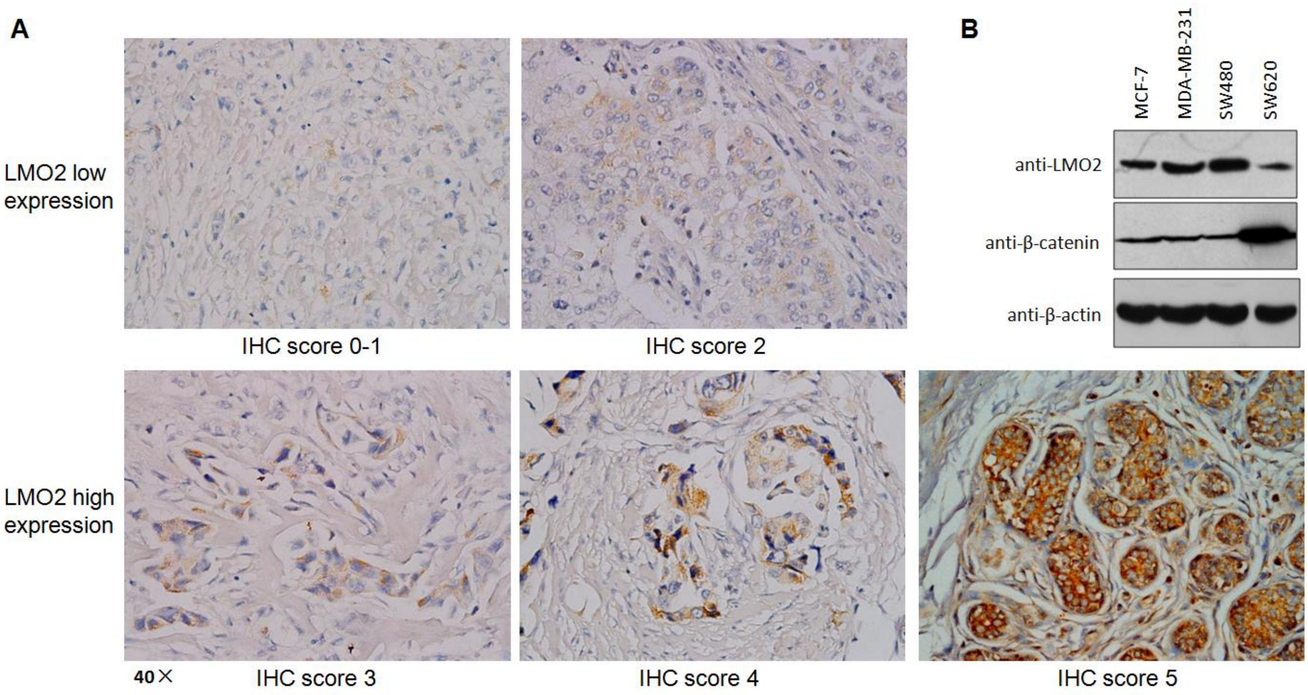

Figure S1. (A) Representative images of anti-LMO2immunohistochemistry staining of breast tissue samplesscored from 0-5. The colorectal tissue samples were also scored based on these criteria. (B) Western blotting images of LMO2 and β-catenin in breast cancer and colorectal cancer cell lines. β-actin was used as the loading control.

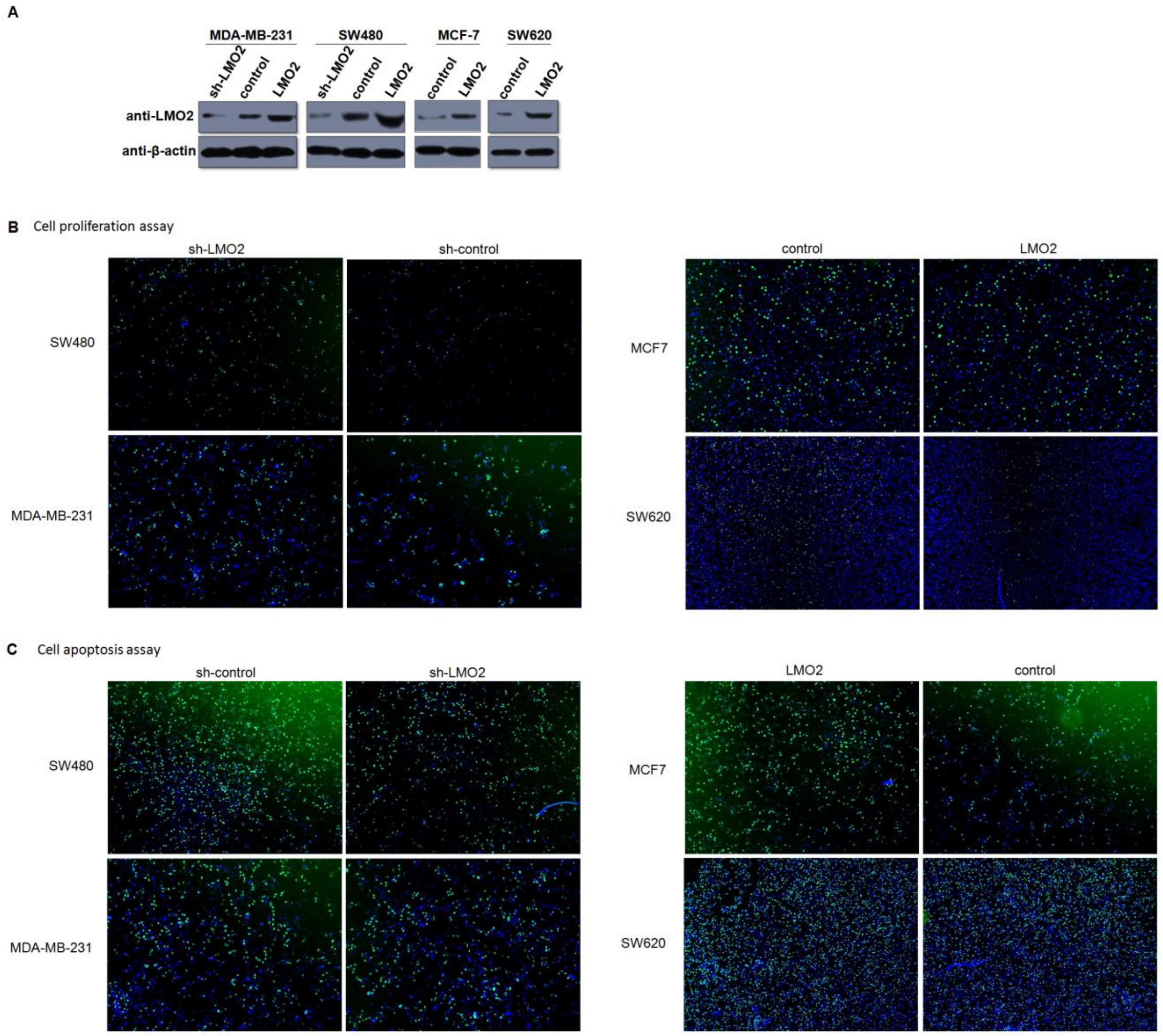

Figure S2. (A) Western blot images confirming LMO2 overexpression and knockdown efficiency in different cancer cell lines. β-actin was used as the loading control. (B) Representative images of Cell-Light™ EdU DNA cell proliferation in the indicated cells. (C) Representative Cell-Light™ EdUTP Apollo®488 TUNEL cell detection assay results. EdU-positive cells were stained with Fluor-488 fluorescence and nuclei were stained with Hoechst 33342. All images were obtained using the Cytation^{TM} 3 system.

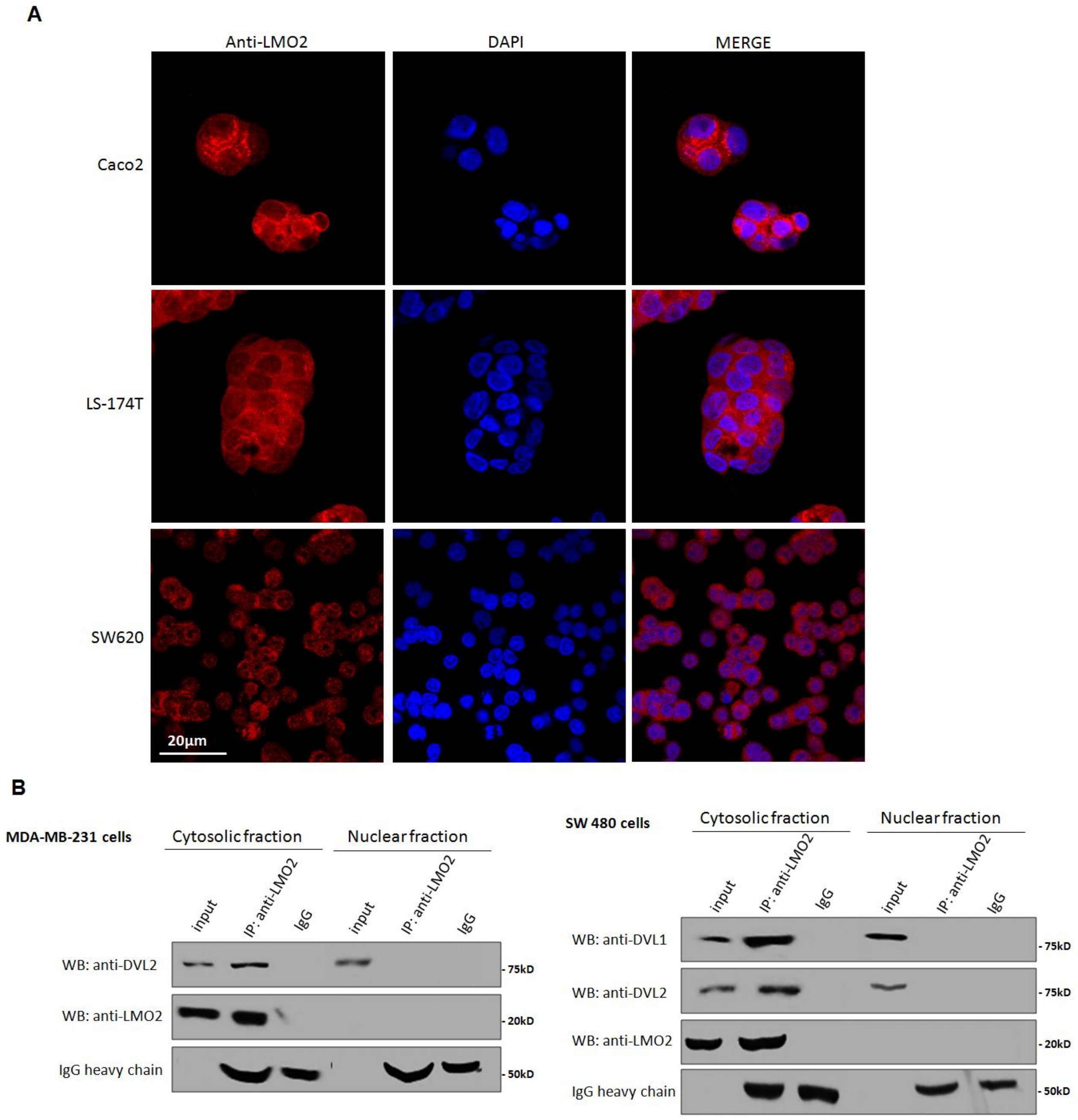

Figure S3. LMO2 interacts with Dishevelled-1/2 primarily in the cytoplasm in breast and colorectal cancer cells. (A) Representative immunofluorescence staining images of LMO2 in Caco-2, LS-174T, and SW620 colorectal cancer cell lines. LMO2 was stained with a mouse anti-LMO2 antibody followed by Alexa fluor-568 secondary fluorescent antibody; the nuclei were stained with DAPI. (B) Co-immunoprecipitation assay to confirm the interaction between DVL-1 or -2 and LMO2 in cytosolic fraction of MDA-MB-231 or

SW480 cells. One milligram of cytosolic protein or 500 μg nuclear protein were used for the co-immunoprecipitation assay, and 1/20 of the cytosolic or nuclear protein from each sample was loaded as the input.

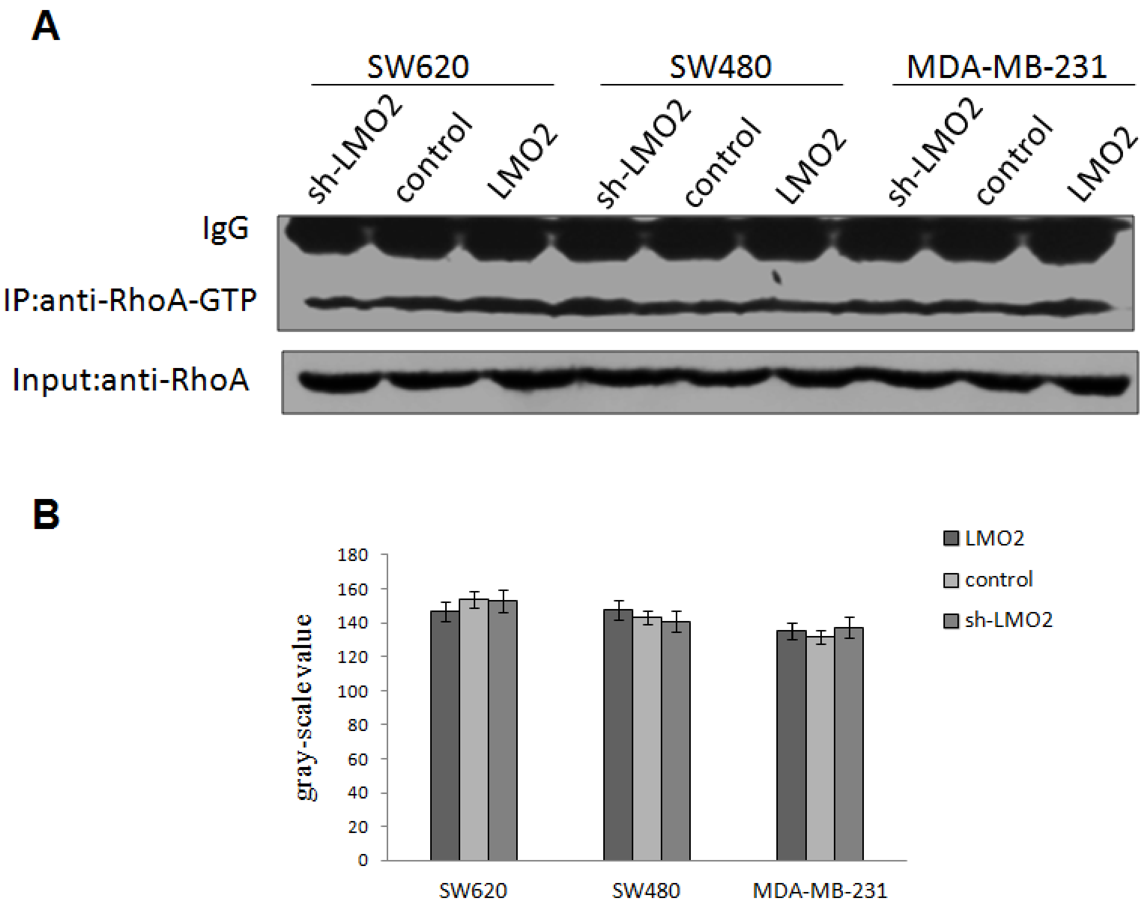

Figure S4. RhoA activity after stimulation with Wnt3A in breast cancer and colorectal cancer cell lines. (A) Western blotting of total and active RhoA in the indicated cells. (B) Quantification of the active RhoA immunoblotting bands. The images were scanned and quantified using ImageJ software. The bars represent the means of three independent experiments and the error bars indicate the standard error.

Original western blot of Figure 3A

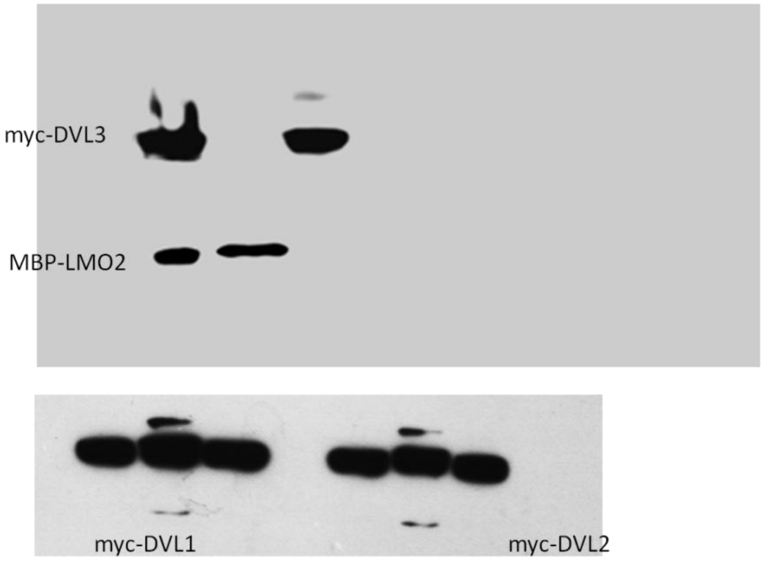

Original western blot of Figure 3B

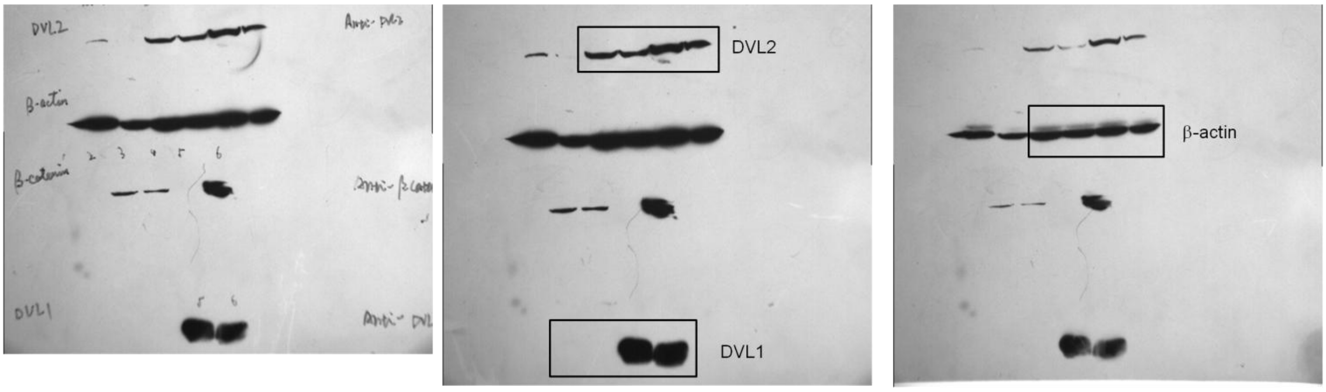

Original western blot of Figure 3C

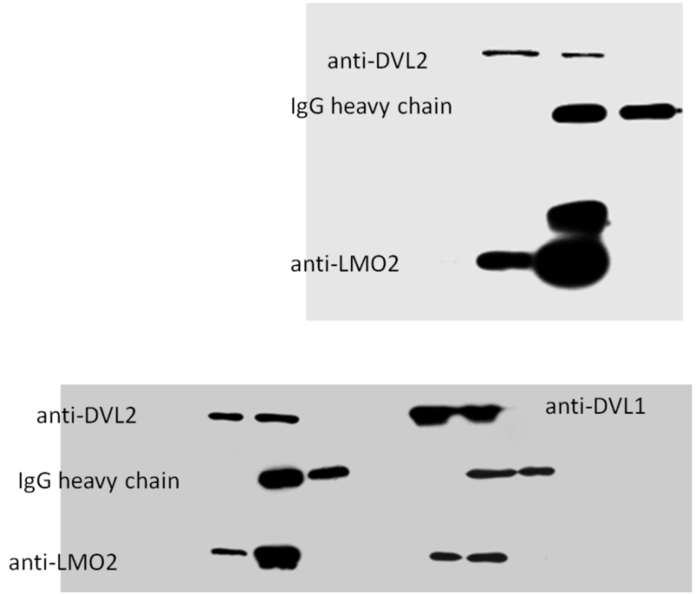

Original western blot of Figure 3E

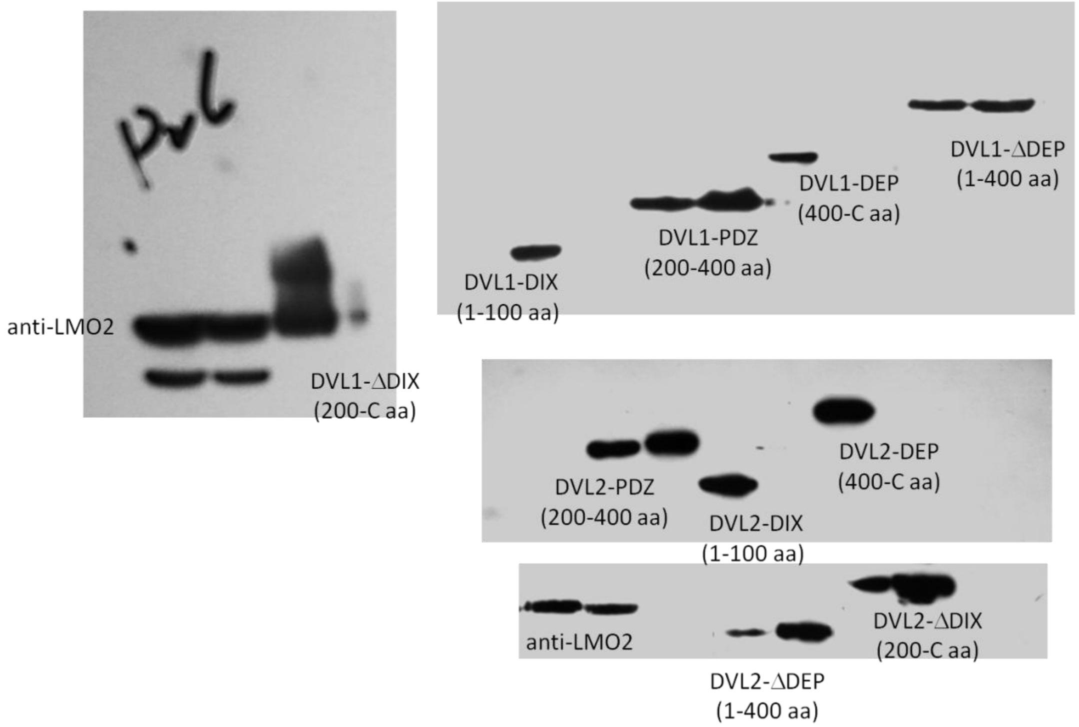

Supplement: Supplementary Information [file srep36050-s2.pdf]
